# Optimizing Communication and Adherence to Iron Chelation Therapy From Diagnosis to Treatment in Patients With Myelodysplastic Syndromes

**Published:** 2016-11-01

**Authors:** Jayshree Shah, Phyllis McKiernan

**Affiliations:** Hackensack University Medical Center, Hackensack, New Jersey

## Abstract

Myelodysplastic syndromes (MDS), a heterogeneous group of blood diseases, are usually diagnosed in older individuals, with a median age at diagnosis of more than 70 years. Anemia is a common symptom in patients with MDS and may require frequent red blood cell transfusions, which can lead to iron overload. Iron chelation therapy is recommended to decrease iron concentrations in tissue and minimize organ dysfunction. However, the currently available iron chelation therapies are associated with side effects, financial constraints, and dosing issues, which may affect patient adherence. Moreover, many patients with MDS lack an understanding of the disease and their prognosis and treatments. This review can be used in the advanced practice setting to discuss the importance of communicating with patients about MDS from the time of diagnosis and will explore strategies to enhance adherence to iron chelation therapy. An individualized approach that weighs the risks and benefits of treatment for older patients with MDS will allow advanced practitioners to set expectations while developing adherence strategies to optimize outcomes. This approach provides a platform for advanced practitioners to communicate with patients to ensure they understand the natural history of MDS, their individual prognoses, and the goals of both active treatment and supportive care.

Melodysplastic syndromes (MDS) refer to a heterogeneous group of blood diseases usually associated with cytopenias (Platzbecker & Adès, 2014; Greenberg et al., 2009; National Comprehensive Cancer Network [NCCN], 2016). It is most often diagnosed in older individuals, with the median age at diagnosis being > 70 years (Ma, Does, Raza, & Mayne, 2007; Sekeres et al., 2008). Although usually indolent, MDS can result in the development of acute myeloid leukemia (AML; Platzbecker & Adès, 2014; NCCN, 2016).

Supportive care is an important component of the management of all patients with MDS (NCCN, 2016). In particular, symptomatic anemia is a common morbidity associated with MDS and requires appropriate supportive care, including red blood cell (RBC) transfusions (NCCN, 2016; Greenberg et al., 2009). However, the frequent use of RBC transfusions may result in iron overload, which is associated with organ dysfunction and increased mortality (Greenberg et al., 2009; NCCN, 2016).

## PATIENT’S UNDERSTANDING OF MDS: DIAGNOSIS AND NATURAL HISTORY

For a patient with MDS, both prognosis and treatment options are determined by his or her prognostic category. This is based on a standard scoring system, such as the International Prognostic Scoring System (IPSS), the revised IPSS (IPSS-R), the World Health Organization classification-based prognostic scoring system (WPSS), or the French-American-British MDS classification (NCCN, 2016; Greenberg et al., 1997, 2012; Malcovati et al., 2007; Bennett et al., 1982). These scores predict a patient’s survival and the likelihood of progressing to AML (NCCN, 2016).

Patients are stratified into two main risk categories: lower risk (IPSS Low or Intermediate-1; IPSS-R Very Low, Low, or Intermediate; WPSS Very Low, Low, or Intermediate) and higher risk (IPSS Intermediate-2 or High; IPSS-R Intermediate, High, or Very High; WPSS High or Very High; NCCN, 2016). For lower-risk patients, the main goal of treatment is hematologic improvement (NCCN, 2016). For higher-risk patients, the main goal of treatment is to alter the natural history of the disease and decrease evolution to AML (NCCN, 2016).

Oncology advanced practitioners (APs) are in a unique position to take the initiative in educating patients about MDS and the potential for developing iron overload related to receiving multiple transfusions over a long period. An Internet survey conducted in March 2009 with patients registered in the Aplastic Anemia and MDS International Foundation (AAMDSIF) (N = 358; median age, 65 years) showed that patients with MDS have a limited understanding of these concepts (Sekeres et al., 2011). More than half of the respondents (55%) did not know their IPSS risk score or category, and only 7% reported having MDS described to them as a cancer (Sekeres et al., 2011).

Two surveys conducted in the United States in 2012 with patients and health-care providers (HCPs) registered in AAMDSIF indicated a lack of concordance between patients with MDS and their HCPs in perceptions about MDS and its treatments (Steensma et al., 2014). For instance, 10% of patients agreed that MDS represented "cancer," compared with 46% of nonphysician HCPs and 59% of physicians (*p* < .001 for both comparisons; Steensma et al., 2014). In addition, 29% of patients vs. 56% of HCPs thought that MDS could be "curable" (*p* < .001; Steensma et al., 2014).

The results of these surveys highlight the disparity between the perceptions and goals of patients and physicians, which in turn can contribute to nonadherence. In a 2014 survey of 16 APs (14 nurse practitioners, 2 physician assistants) who were managing patients with lower-risk MDS, 81% indicated there was a need for more patient education about the disease, particularly with regard to adverse events, time to treatment response, compliance, and MDS as a cancer (Kurtin, Latsko, & Finley-Oliver, 2016). As APs, we have the opportunity to reeducate patients and caregivers about how MDS is a heterogeneous disease that evolves through intrinsic and extrinsic factors (the characteristics of the malignant clone and the bone marrow microenvironment, respectively; Kurtin, 2011). The development of malignancy is accompanied by symptomatic anemia, bleeding, and an increased risk of infections due to the decline in bone marrow function (Kurtin, 2011).

Informing and involving patients in the management of MDS are key to ensuring individualized therapy and maximizing patient satisfaction (Smith, 2012). Most patients diagnosed with MDS are elderly (Ma et al., 2007; Sekeres et al., 2008) and may have comorbidities that affect the management of their disease and their treatment options (Platzbecker & Adès, 2014; NCCN, 2016; Kurtin, 2010). Functional status and frailty are additional factors that influence the treatment of elderly patients but may be difficult for HCPs to assess in the office setting (Kurtin, 2010).

It is particularly important to discuss and manage treatment expectations (Smith, 2012). Quality of life, which is often compromised in patients with MDS, also needs to be addressed (Sekeres et al., 2011; NCCN, 2016). Advanced practitioners should understand that patient preferences for involvement in decision-making might vary. In a prospective cohort study in patients with newly diagnosed higher-risk MDS (N = 280; mean age, 70 years), nearly half (47%) preferred to take a more passive role (Efficace et al., 2014). Patients with lower hemoglobin levels and worse health-related quality of life had a greater likelihood of preferring a more passive role (Efficace et al., 2014). Moreover, the study found that older patients and those with less education were less likely to request prognostic information (Efficace et al., 2014).

## ANEMIA AND IRON OVERLOAD

Anemia and other cytopenias are a hallmark of MDS. Approximately 90% of patients with MDS have anemia at diagnosis, and leukocyte-reduced RBC transfusions are an important part of supportive care for these patients (Greenberg et al., 2009; NCCN, 2016). The National Comprehensive Cancer Network Clinical Practice Guidelines in Oncology (NCCN Guidelines) for Myelodysplastic Syndromes note that although RBC transfusions are recommended for symptomatic anemia, the units transfused should be minimized in noncardiac patients, according to the American Society of Hematology (ASH) Choosing Wisely recommendations for the appropriate use of hematologic tests and treatments (NCCN, 2016; Hicks et al., 2013). The Choosing Wisely campaign, led by the nonprofit American Board of Internal Medicine Foundation in conjunction with professional societies, aims to encourage dialogue between physicians and patients about the appropriate use of medical care, including its costs and benefits (Hicks et al., 2013). The guiding principles for Choosing Wisely are listed in Table 1, and a full list of the current ASH recommendations can be found online at http://www.choosingwisely.org/societies/american-society-of-hematology/.

**Table 1:**
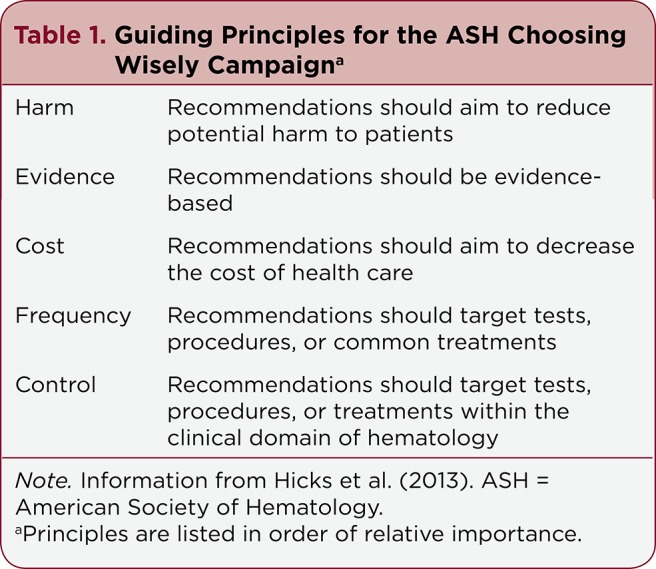
Guiding Principles for the ASH Choosing Wisely Campaign

In addition, daily iron chelation therapy should be considered to decrease iron overload for patients who have received more than 20 to 30 RBC transfusions (Figure), particularly for lower-risk patients (IPSS: Low/Intermediate-1) and potential transplant patients (NCCN, 2016). For patients who have a serum ferritin level > 2,500 ng/mL, a target ferritin level of < 1,000 ng/mL is recommended (NCCN, 2016). It is an important goal to review this information with patients, who might otherwise take a passive approach and not understand the implications of developing iron overload over time. Phlebotomy may be an option for patients who have a hematologic response to MDS treatment (Shah, Kurtin, Arnold, Lindroos-Kolqvist, & Tinsley, 2012). Since patients with higher-risk MDS have a shorter estimated survival, iron chelation therapy is generally not warranted because of the reduced risk of long-term toxicities related to iron overload (Shah et al., 2012). It should be noted that there is ongoing controversy about the utility of iron chelation therapy in patients with MDS; European guidelines classify their recommendations for iron chelation therapy in MDS as having the lowest level of evidence (Fenaux, Haase, Sanz, Santini, & Buske, 2014; Malcovati et al., 2013).

**Figure 1:**
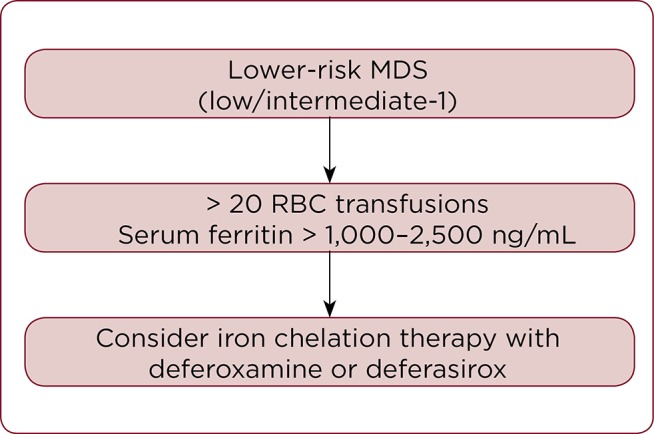
Daily iron chelation therapy should be considered to decrease iron overload for patients who have received more than 20 to 30 red blood cell (RBC) transfusions, particularly for lower-risk patients with myelodysplastic syndromes (MDS) and potential transplant patients. Information from NCCN (2016); Fenaux et al. (2014); Malcovati et al. (2013).

## OPTIMIZATION OF IRON CHELATION THERAPY

Three iron chelation therapies are available in the United States: deferasirox (Exjade, Jadenu [Novartis Pharmaceuticals Corporation, 2016a, 2016b]); deferoxamine (Desferal [Novartis Pharmaceuticals Corporation, 2011]); and deferiprone (Ferriprox [ApoPharma USA, 2015]; Table 2). The NCCN Guidelines panel recommends either subcutaneous deferoxamine or oral deferasirox for lower-risk patients with MDS (NCCN, 2016). Iron chelation therapy aims to decrease iron concentrations in tissue to safe levels and promote a negative iron balance (Porter, 2001). Iron chelators meditate their effects by stably binding the six coordination sites on the iron atom to detoxify it and prevent redox cycling and subsequent oxidative damage (Porter, 2001).

**Table 2:**
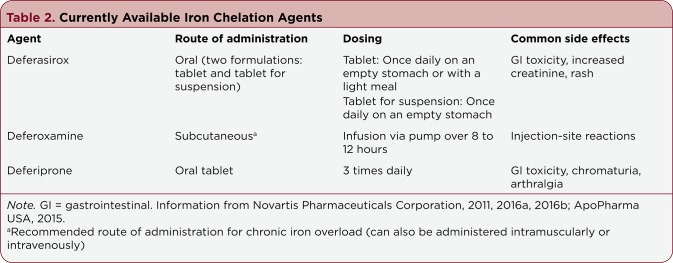
Currently Available Iron Chelation Agents

Although no randomized, controlled trials of iron chelation therapy in patients with MDS have demonstrated an impact on mortality, a meta-analysis of eight observational trials indicated an association between iron chelation and improved survival (Mainous, Tanner, Hulihan, Amaya, & Coates, 2014). Of the eight trials, seven included only low-risk patients, indicating the survival benefit seen in the meta-analysis (an average increase of 5 years) may be limited to patients with lower-risk MDS (Mainous et al., 2014).

There is stronger evidence that iron chelation therapy improves hematologic parameters in patients with MDS. In a post-hoc analysis from the phase IIIb Evaluation of Patients’ Iron Chelation with Exjade (EPIC) trial, the use of deferasirox in patients with MDS (N = 341) for 1 year was associated with improved erythroid responses (either a reduced need for transfusions or increased hemoglobin levels) in 21.5% of patients, improved platelet responses in 13.0% of patients, and improved neutrophil responses in 22.0% of patients (Gattermann et al., 2012a).

**Deferasirox**

There are two formulations of deferasirox: a once-daily tablet for oral suspension, which must be dispersed in liquid (water or juice) and taken on an empty stomach (Exjade), and a once-daily tablet approved in 2015, which can be taken either on an empty stomach or with a low-fat meal (Jadenu; Novartis Pharmaceuticals Corporation, 2016a, 20156b). The newer formulation allows for simplified dosing compared with the older formulation (Novartis Pharmaceuticals Corporation, 2016a, 20156b).

Deferasirox is contraindicated in patients with high-risk MDS and a poor performance status (Novartis Pharmaceuticals Corporation, 2016a, 20156b). It is also contraindicated in patients with thrombocytopenia (platelet counts < 50,000/mL), one of the cytopenias common in patients with MDS (Kantarjian et al., 2007), and in patients with decreased renal function (serum creatinine > 2× the age-appropriate upper limit of normal or creatinine clearance < 40 mL/min; Novartis Pharmaceuticals Corporation, 2016a, 2016b).

Elderly patients need to be monitored closely for toxicity, as there is a greater frequency of decreased hepatic, renal, and/or cardiac function in this population; dose selection should start with lower doses (Novartis Pharmaceuticals Corporation, 2016a, 2016b). In addition, treatment interruption should be considered once serum ferritin levels fall below 500 ng/mL or if there is auditory or ocular toxicity (Novartis Pharmaceuticals Corporation, 2016a, 2016b). In elderly patients, including those with MDS, the decision to use an iron chelator should be individualized based on patient considerations and the risks and benefits of treatment (Novartis Pharmaceuticals Corporation, 2016a, 2016b).

The most common side effects in patients receiving deferasirox in clinical trials were gastrointestinal (GI) adverse events (AEs), skin rash, and increases in serum creatinine (Novartis Pharmaceuticals Corporation, 2016a, 2016b). There is also a risk of hepatic toxicity, which may be fatal; hearing disorders, including high-frequency hearing loss and decreased hearing; and eye disorders, including lens opacities, cataracts, elevated intraocular pressure, and retinal disorders (Novartis Pharmaceuticals Corporation, 2016a, 2016b). Patients should undergo testing of the following parameters before and during iron chelation therapy to monitor for these toxicities: granulocyte and serum creatinine levels and physical examinations monthly; serum ferritin levels every 3 months; and auditory, liver and myocardiac iron stores (T2* magnetic resonance imaging [MRI]), and ophthalmic testing annually (Shah et al., 2012).

Deferasirox has demonstrated efficacy at reducing iron overload, as measured by liver iron concentration in clinical trials in transfused patients with anemias, including â-thalassemia, sickle cell disease (SCD), and MDS (Cappellini et al., 2006; Piga et al., 2006; Vichinsky et al., 2007; Porter et al., 2008; Taher et al., 2009). Beta-thalassemia was chosen as the model disease investigated in the pivotal phase III trial, and long-term efficacy and safety have been demonstrated for up to 5 years of follow-up in this patient population (Cappellini et al., 2006, 2011).

Patients with SCD were evaluated in a separate phase II trial (Vichinsky et al., 2007); a placebo-controlled phase II trial of deferasirox oral suspension in lower-risk patients with MDS with transfusional iron overload has completed enrollment, and results are pending (TELESTO; NCT00940602). The large EPIC study was performed in 1,744 patients with different types of anemia (thalassemia, SCD, MDS, and others) and showed reductions in serum ferritin with deferasirox using a dosing schema based on iron intake via transfusions, followed by subsequent dose adjustments based on serum ferritin levels (Cappellini et al., 2010).

**Deferoxamine**

The standard recommended administration method for deferoxamine is slow subcutaneous infusion over 8 to 12 hours; it can also be given via intramuscular or intravenous administration (Novartis Pharmaceuticals Corporation, 2011). There may be a higher risk of eye disorders, including vision impairments, optic neuritis, cataracts, corneal opacities, and retinal pigment abnormalities in elderly patients taking deferoxamine, although it is not clear whether it is dose related (Novartis Pharmaceuticals Corporation, 2011). Periodic eye evaluations, including visual acuity tests, slit-lamp examinations, and funduscopy, are recommended for patients undergoing long-term treatment with deferoxamine (Novartis Pharmaceuticals Corporation, 2011). Most reports of ocular toxicity have occurred in patients taking high doses of deferoxamine for an extended period or in patients with low levels of serum ferritin, and it is usually reversible after stopping treatment (Novartis Pharmaceuticals Corporation, 2011).

As with deferasirox, dose selection for elderly patients should start at the low end of the dosing range, reflecting the greater frequency of decreased hepatic, renal, or cardiac function and of concomitant diseases or medications (Novartis Pharmaceuticals Corporation, 2011).

**Deferiprone**

Deferiprone, an oral agent approved for the treatment of transfusional iron overload due to thalassemia when current treatment is not adequate (ApoPharma USA, 2015), is not recommended by the NCCN Guidelines panel for the treatment of iron overload in patients with MDS (NCCN, 2016).

## ADHERENCE TO IRON CHELATOR THERAPY

There are distinct adherence challenges associated with the different formulations of the iron chelators recommended for patients with MDS. Subcutaneous deferoxamine is associated with injection-site reactions, including pain, bruising, swelling, and infections (Hoffbrand, Taher, & Cappellini, 2012; Porter, Evangeli, & El-Beshlawy, 2011; Novartis Pharmaceuticals Corporation, 2011). Moreover, older patients may have difficulty adjusting to the infusion pumps needed to deliver a continuous subcutaneous infusion of deferoxamine (Porter et al., 2011).

The oral drug deferasirox is associated with GI side effects, including abdominal pain, diarrhea, nausea, and vomiting, potentially compromising adherence (Porter et al., 2011; Novartis Pharmaceuticals Corporation, 2016a, 2016b). It should be noted that the incidence of diarrhea with deferasirox in clinical trials is roughly two to four times higher in patients with MDS than in patients with thalassemia or SCD (Cappellini et al., 2010; Novartis Pharmaceuticals Corporation, 2016a, 2016b).

A study conducted by the Thalassemia Clinical Research Network investigated adherence to iron chelation therapy in a cohort of patients with thalassemia, with 79 patients taking deferoxamine and 186 patients taking deferasirox. Patients with poorer adherence cited injection-site pain, sores, and saturation of injection sites as barriers to iron chelation therapy with deferoxamine and abdominal pain and bad taste in the mouth as barriers with deferasirox (Trachtenberg et al., 2011). Patients in the study who had previously taken other iron chelators reported that deferasirox was easier to use and, according to some patients, was associated with better adherence than deferoxamine (Trachtenberg et al., 2011).

Another study used semistructured interviews of patients and caregivers (including three patients with MDS) to evaluate factors that may have an impact on adherence to iron chelation therapy (Bal, Cote, Lasch, & Huang, 2014). Reasons why patients were adherent to iron chelators included perceived health and longevity benefits, clinician and caregiver support, and an established routine for taking medication (Bal et al., 2014). Reasons why patients were nonadherent included palatability issues, the texture and solubility of the formulation, GI side effects, and food restrictions for dosing (Bal et al., 2014). The effect of GI toxicity on medication taking was also seen in larger clinical trials with deferasirox. In both the prospective, multicenter EPIC study and the observational eXtend and eXjange studies, the primary reason for discontinuation of deferasirox for patients with MDS was AEs, particularly GI AEs (Gattermann et al., 2010, 2012b).

Nevertheless, patients taking deferasirox had high levels of adherence and persistence. In the EPIC study, in which patients with or without prior iron chelator use were initiated on deferasirox tablets for oral suspension, patient-reported adherence to deferasirox was more than 80% for patients with MDS (85.7% for patients with prior iron chelator use; 82.9% for patients without prior iron chelator use; Porter et al., 2012). For patients with MDS who were using an iron chelator (either deferoxamine or deferiprone) before the study, adherence to the prior iron chelator was only 62.5% at the start of the study (Porter et al., 2012). The level of persistence (defined as never thinking about stopping therapy) to deferasirox was 77.1% for patients with MDS who did not previously use an iron chelator (Porter et al., 2012). For patients with MDS who had previously used an iron chelator, there was a slight decrease in persistence upon starting deferasirox compared with the prior therapy (75.9% with prior iron chelators at baseline; 69.0% with deferasirox during the study; Porter et al., 2012).

An expert panel discussion of hematology/oncology physicians conducted in July 2014 produced a number of recommendations for the prevention and management of GI toxicity in patients with MDS taking deferasirox (Nolte et al., 2015). As AEs due to GI toxicity are a key barrier to adherence, APs should inform patients about the potential for GI side effects prior to starting deferasirox (Nolte et al., 2015). Gastrointestinal toxicity can be addressed through optimized dosing schedules, namely starting at a low dose (a flat dose of 500 mg once daily) and taking the dose before the evening meal (Nolte et al., 2015). Although neither of these recommendations is supported by evidence from clinical trials, the members of the panel reasoned that taking the dose in the evening rather than in the morning might shift the occurrence of GI effects to nighttime, thereby minimizing the disruption of patients’ daily routines (Nolte et al., 2015).

The panel also created algorithms and tables with specific recommendations for managing diarrhea, abdominal pain, and nausea/vomiting (Nolte et al., 2015). It is not known whether the newer tablet formulation of deferasirox will improve tolerability compared with the tablet for oral suspension (Nolte et al., 2015). Unlike the oral suspension medication, the tablet formulation does not contain lactose or sodium lauryl sulfate—two components that may play a role in GI toxicity (Nolte et al., 2015). Also, the ability to take the tablet with a low-calorie, low-fat meal may help promote adherence to treatment (Nolte et al., 2015). This approach may benefit patients who may otherwise be in the habit of eating a meal before taking any prescribed medication. Promoting a daily routine of eating a low-calorie, low-fat meal and taking the new tablet formulation of deferasirox may result in a positive outcome, with increasing overall adherence.

Adherence to iron chelator therapy may help to lessen the disease burden associated with MDS. In a retrospective study evaluating MDS in US Medicare beneficiaries over a 3-year period (2003–2005), comorbidities, including cardiac events, diabetes, dyspnea, liver disease, and infections, were significantly more common in patients with MDS than in the general Medicare population (Goldberg et al., 2010). Moreover, patients with MDS receiving RBC transfusions had a significantly greater prevalence of cardiac events, dyspnea, and infections compared with nontransfused patients with MDS (Goldberg et al., 2010). Age-adjusted mortality was also significantly higher in patients with MDS vs. the overall Medicare population and in transfused vs. nontransfused patients with MDS (Goldberg et al., 2010). The economic impact of MDS was substantial, with significantly higher Medicare costs for patients with MDS vs. the overall Medicare population in 2003 (median $16,181 vs. $1,575; *p* < .001), 2004 (median $9,703 vs. $1,772; *p* < .001), and 2005 (median $6,872 vs. $1,912; *p* < .001; Goldberg et al., 2010).

Few studies have investigated the relation between adherence to iron chelation therapy and outcomes for patients with MDS. However, there is some evidence that adherence to iron chelation may reduce health-care utilization and costs. In a study of patients with SCD in a Medicaid population in six different U.S. states, adherent patients had lower total costs and SCD-specific costs than did nonadherent patients, mainly due to lower inpatient costs (Vekeman et al., 2014). In that study, adherence to iron chelation therapy, as measured by medication possession ratio (MPR), was low (48.3% had an MPR ≥ 0.80), but adherence to deferasirox was higher than adherence to deferoxamine (mean MPR = 0.75 vs. 0.68; *p* < .05; Vekeman et al., 2014). Additional studies are needed to determine the effect of adherence to iron chelators on clinical and other outcomes.

## INTERVENTIONS TO IMPROVE ADHERENCE

A patient’s adherence to medication is influenced by many factors. Barriers to adherence are listed in Table 3 (Osterberg & Blaschke, 2005; Ho, Bryson, & Rumsfeld, 2009). Even patients with cancer may have poor adherence to their prescribed medications, and APs should encourage medication self-management strategies and screen their patients for potential barriers to adherence (Faiman, 2011).

**Table 3:**
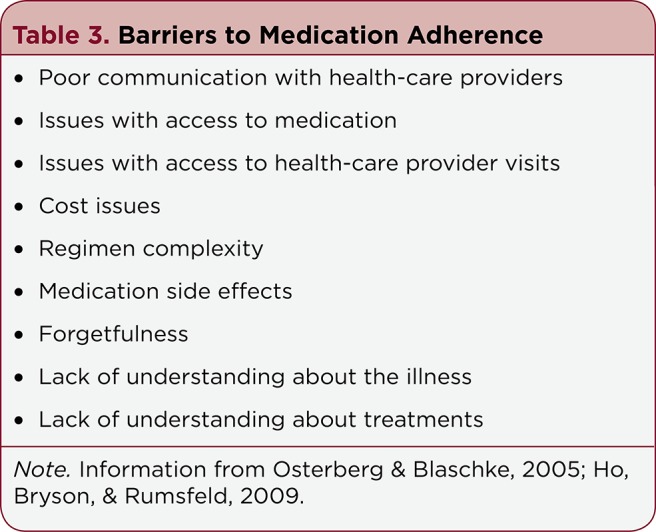
Barriers to Medication Adherence

Although numerous strategies have been developed to promote adherence, the use of behavioral interventions to improve medication adherence is an approach supported by data from clinical trials. In US adults with chronic conditions, patient education with behavioral support was shown to be effective in improving adherence, according to a systematic review from the Agency for Healthcare Research and Quality (Viswanathan et al., 2012).

Similarly, a meta-analysis of 33 randomized, controlled trials supported a role for interventions that focus on behavioral strategies, such as decreasing the number of doses or using prompts or devices to dispense medication, for older adults (Conn et al., 2009). The meta-analysis also found that written instructions appeared to be more helpful than verbal instructions for older adults (Conn et al., 2009). These strategic interventions support the expectations set by APs who may be reviewing the goals of chelation therapy with their MDS patients.

Patient-centered care, including taking patient preferences into account through shared decision-making, is another important concept in the care of all patients, including those with MDS (Barry & Edgman-Levitan, 2012; Oshima Lee & Emanuel, 2013). Shared decision-making was first introduced in 2001 in the report "Crossing the Quality Chasm: A New Health System for the 21st Century" from the Institute of Medicine (2001), which recommends patients receive appropriate information so they may exercise control over their care. It is believed that taking a more active role in their care may help to enhance patient adherence (Viswanathan et al., 2012; Barry & Edgman-Levitan, 2012; Oshima Lee & Emanuel, 2013). If there is an opportunity for caregivers and other family members to participate in the shared decision-making concerning iron chelation, it gives the patient more reinforcement to achieving adherence. However, as previously noted, some patients with MDS prefer a less active role (Efficace et al., 2014), and this also needs to be taken into account with respect to adherence to iron chelator therapy.

## DISCUSSION

A complicated heterogeneous type of malignancy, MDS results in cytopenias associated with the development of anemia, neutropenia, and thrombocytopenia. Patients with MDS and anemia often require repeated RBC transfusions, which can potentially lead to iron overload. Iron chelation therapy may have a survival benefit for low-risk MDS patients, but nonadherence to therapy, due to barriers such as GI toxicities and access to medication and health care, may impact outcomes.

Improving medication adherence has been a focus for APs, as the use of oral agents for cancer treatment and supportive care has been increasing. It is essential to communicate to patients about the natural history of MDS, their prognosis, and the goals of treatment, as well as any anticipated side effects. Resources that may be helpful for APs and their patients are listed in Table 4. Recommended testing should be performed prior to the start of chelation therapy and throughout therapy (Shah et al, 2012).

**Table 4:**
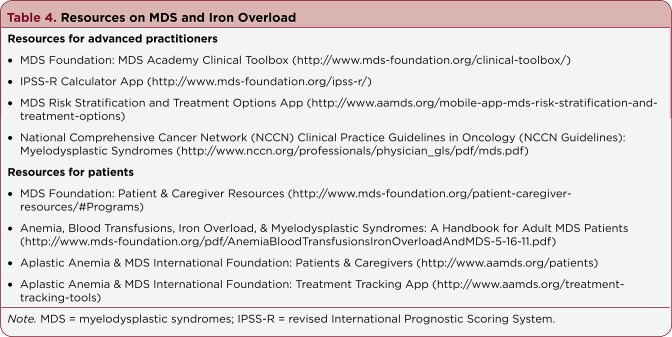
Resources on MDS and Iron Overload

As the majority of patients with MDS are elderly, simplified dosing and/or newer formulations may help to promote adherence to iron chelation therapy, with potentially fewer side effects. Providing patient and caregiver education with behavioral support is an effective strategy for increasing adherence in adults with chronic conditions, including older adults. Reviewing the side-effect profile prior to starting iron chelation and monitoring throughout therapy are also important to improving adherence. These actions allow further research to be done to evaluate the effects of new formulations for patients with MDS who experienced side effects with older formulations, resulting in poor adherence.

Although there is little evidence to support specific nursing interventions to improve adherence (Tipton, 2015), APs are in a unique position to identify patients at risk for nonadherence and to recognize the time to intervene (Spoelstra & Sansoucie, 2015). Using a patient-centered approach and encouraging communication with HCPs are ways for APs to impact adherence to iron chelation therapy in patients with MDS.

**Acknowledgments**

Novartis Pharmaceuticals Corporation provided financial support for medical editorial assistance. Susan DePetris, PhD, of Phase Five Communications, provided medical editorial assistance.
